# The economic value of mismatch repair–guided immunotherapy in endometrial cancer: companion testing and patient selection

**DOI:** 10.3389/fmed.2026.1951624

**Published:** 2026-09-09

**Authors:** Sihan Jing, Yuan He, Yang Liu

**Affiliations:** 1 School of Maritime Economics and Management, Dalian Maritime University, Dalian, China; 2 Postdoctoral Research Station, China Minsheng Bank, Beijing, China; 3 Postdoctoral Research Station, School of Finance, Renmin University of China, Beijing, China; 4 Department of Obstetrics and Gynecology, Xijing Hospital of Fourth Military Medical University, Xi'an, Shaanxi, China

**Keywords:** companion diagnostics, cost-effectiveness, endometrial cancer, immunotherapy, microsatellite instability, mismatch repair, patient selection, value-based care

## Abstract

Mismatch repair (MMR) status has moved from hereditary-cancer screening to a central predictive biomarker for immunotherapy in endometrial cancer. The marked difference in treatment benefit between MMR-deficient/microsatellite instability-high (dMMR/MSI-H) and MMR-proficient/microsatellite-stable (pMMR/MSS) tumors creates an economic rationale for companion testing, but the value of testing depends on assay accuracy, treatment price, duration, survival extrapolation, and local willingness-to-pay thresholds. This Mini Review examines the clinical and economic value of MMR-guided immunotherapy in advanced and recurrent endometrial cancer. A targeted literature search of PubMed/MEDLINE, Embase, Web of Science, and the Cochrane Library from database inception through 31 July 2026. We summarize practical testing pathways based on four-protein immunohistochemistry, reflex mutL homolog 1 (MLH1) promoter methylation, MSI assays, and selective next-generation sequencing; review MMR-stratified evidence from major immunotherapy trials; and compare published cost-effectiveness analyses of pembrolizumab, dostarlimab, atezolizumab, durvalumab-based regimens, and pembrolizumab plus lenvatinib. Across studies, dMMR/MSI-H disease generally generates the largest quality-adjusted survival gains and the most favorable incremental cost-effectiveness ratios, whereas the economic case in pMMR/MSS disease is more sensitive to drug pricing and modeling assumptions. Overall, MMR testing can function as an allocation technology that prioritizes high-value treatment, while future work should harmonize testing, manage discordant results, refine biomarkers for pMMR tumors, and incorporate long-term survival, quality of life, equity, and value of information into economic models.

## 1 Introduction

Endometrial cancer is the most common gynecologic malignancy in many high-income settings, and its incidence and mortality are increasing in parallel with population aging and metabolic risk factors (1–3). Molecular classification has changed the disease from a largely histology-based entity into four biologically distinct groups: POLE-ultramutated, MMR-deficient, p53-abnormal, and no specific molecular profile (4–8). Among these, MMR status is the most immediately actionable because it informs Lynch syndrome evaluation, prognosis, and the probability of response to immune checkpoint inhibition (5, 6). MMR status denotes the functional integrity of the DNA mismatch-repair pathway, which corrects base-base mismatches and small insertion-deletion loops generated during DNA replication; loss of this function promotes microsatellite instability, mutational accumulation, and an immunogenic phenotype (9–11).

The clinical meaning of MMR status has expanded rapidly. Randomized phase III trials have established anti–PD-1 or anti–PD-L1 therapy combined with carboplatin-paclitaxel as an effective first-line strategy for advanced or recurrent disease, with the largest relative and absolute effects consistently observed in dMMR/MSI-H tumors (12–18). Yet immunotherapy is expensive, treatment may continue for two or three years, and broad regulatory indications can expose large pMMR populations to substantial budget impact for smaller average gains. The economic value of an MMR test therefore extends beyond its laboratory price: it changes the expected benefit distribution, the opportunity cost of treatment, and the design of reimbursement policies.

This Mini Review evaluates MMR-guided immunotherapy as a linked diagnostic–therapeutic strategy. It focuses on three questions: how companion testing creates value; why published cost-effectiveness findings differ across regimens and jurisdictions; and how patient selection can evolve beyond a binary dMMR/pMMR classification while remaining implementable in routine care.

## 2 Why MMR status creates economic value

**Figure 1:**
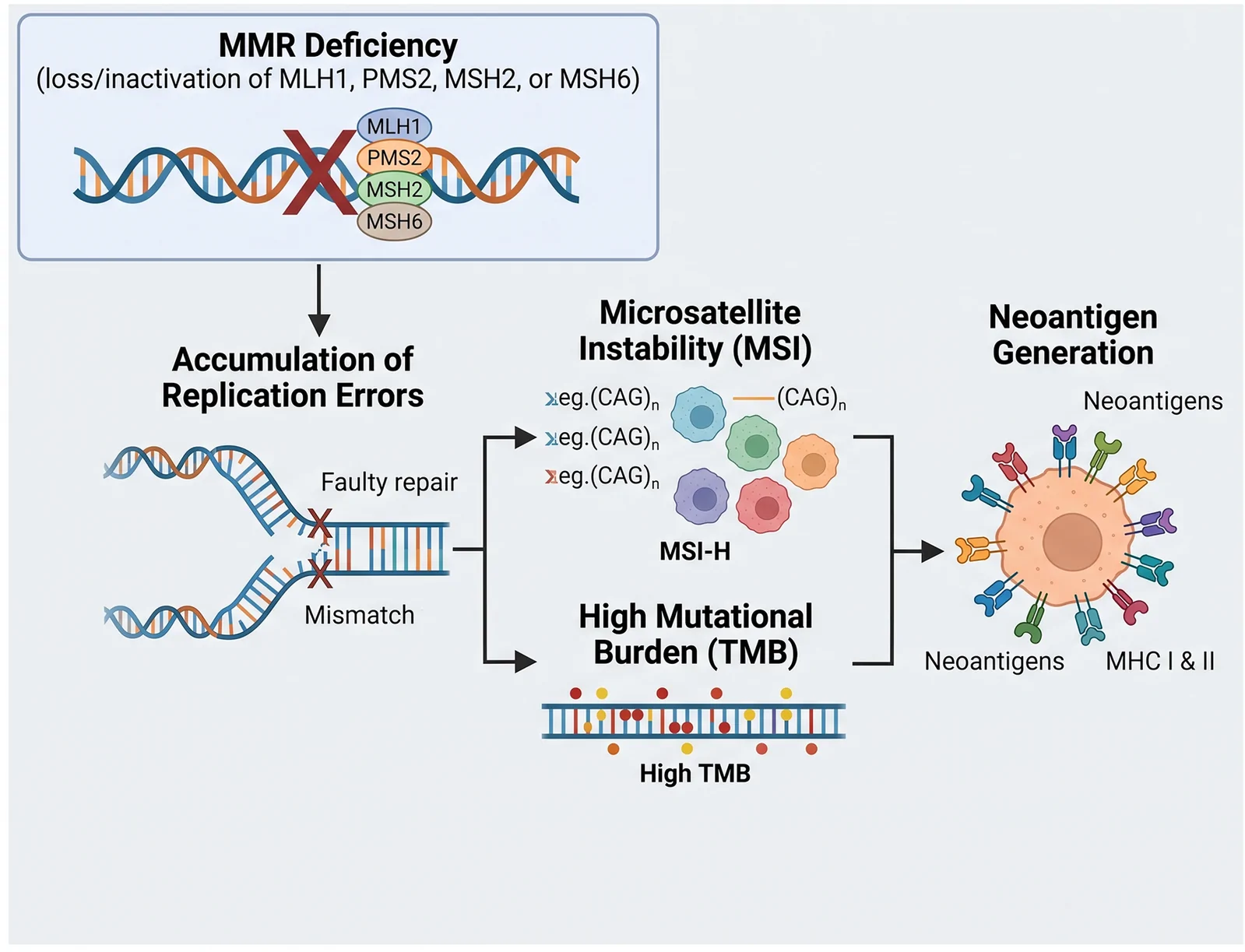
Mechanistic consequences of mismatch repair deficiency. Loss of MLH1, PMS2, MSH2, or MSH6 causes replication-error accumulation, resulting in microsatellite instability, high tumor mutational burden (TMB), and increased neoantigen generation.

MMR deficiency results from loss or inactivation of MLH1, PMS2, MSH2, or MSH6. Accumulation of replication errors generates microsatellite instability, high tumor mutational burden (TMB), neoantigens, interferon signaling, and lymphocyte recruitment (Figure 1). This biology explains why dMMR/MSI-H tumors are disproportionately sensitive to PD-1 blockade (9–11, 19–21). Economically, MMR status behaves as a treatment-effect modifier. A biomarker has high allocative value when it separates patients with large incremental benefit from those with smaller or uncertain benefit, especially when the intervention has high acquisition and toxicity-management costs.

The magnitude of this heterogeneity is clinically material. In NRG-GY018, pembrolizumab plus chemotherapy markedly reduced progression or death in dMMR disease and also improved progression-free survival in pMMR disease (12). An updated analysis continued to favor pembrolizumab in both cohorts, although overall-survival estimates remained less mature (13). In RUBY, dostarlimab showed a similarly large and durable effect in dMMR/MSI-H disease (14), and an updated analysis later reported overall-survival benefit in the overall population with a particularly low hazard ratio in the dMMR subgroup (15). AtTEnd and DUO-E further confirmed that MMR status identifies major heterogeneity in benefit from checkpoint-based combinations (16, 17). An extracted individual patient data meta-analysis estimated 3-year absolute gains of 36% in progression-free survival and 28% in overall survival for dMMR tumors, compared with a 6% absolute progression-free-survival gain in pMMR tumors (18).

These differences translate directly into health-economic outcomes. When the incremental QALY gain is large, the cost of testing becomes negligible relative to the value of selecting treatment. Conversely, when average benefit is modest, small changes in price, duration, utilities, or survival extrapolation can move the ICER above or below a payer's threshold. MMR-guided treatment is therefore best understood as a portfolio decision rather than a single drug decision: testing, treatment, monitoring, and downstream care must be evaluated together.

## 3 Companion testing as an economic intervention

Four-protein MMR immunohistochemistry is a practical first-line assay because it is relatively inexpensive, widely available, and indicates the likely affected gene pair. Current professional guidance and test-strategy recommendations support MMR assessment in all endometrial cancers (4–6, 22). Loss of MLH1/PMS2 should generally trigger reflex MLH1 promoter methylation testing to distinguish the common sporadic epigenetic mechanism from cases that require germline evaluation. MSI-PCR or NGS can confirm equivocal findings, resolve discordance, and simultaneously provide TMB and other therapeutic information (9–11, 23).

Testing creates value through several channels. First, it provides allocative value by identifying patients with the highest expected immunotherapy benefit. Second, testing at initial diagnosis produces intertemporal value: results are available when advanced or recurrent treatment decisions arise, avoiding repeat biopsy and treatment delay. Third, MMR testing has familial value through Lynch syndrome detection. These benefits must be balanced against assay costs, pathology workload, turn-around time, and follow-up testing. A Canadian appraisal found that reflex MLH1 methylation consumed substantial resources because most MLH1/PMS2-deficient tumors were methylated (23), illustrating the need to examine local prevalence and workflow rather than assume that every additional assay has positive net value.

Assay failure can destroy value. Endometrial cancers may show subclonal loss, heterogeneous staining, isolated MSH6 loss, low-level MSI, or discordance between IHC and molecular assays. Discordance was reported in 3.8% of a 666-case series (11); pathology review explained many cases and revealed clinically relevant subclonal or heterogeneous patterns. Misclassifying a true dMMR tumor can deny high-value treatment, whereas false-positive classification can generate low-value exposure to costly therapy. For equivocal cases, expert review and orthogonal testing should therefore be viewed as economic safeguards, not merely technical refinements.

The optimal testing breadth also depends on the comparator. In stage III disease, full tumor molecular testing could be cost-saving compared with no molecular testing but economically unfavorable compared with MMR IHC alone (24). A 2026 analysis similarly suggested that MMR/p53 IHC with selective reflex NGS produced more timely information with less unnecessary sequencing than universal upfront NGS (25). These studies support a tiered strategy: universal low-cost MMR testing, targeted methylation or MSI confirmation, and selective broader sequencing when results can alter therapy or trial eligibility.

## 4 Economic evidence for MMR-stratified immunotherapy

Published economic evaluations do not produce a single universal conclusion, but they consistently identify MMR status and drug price as major determinants. A U.S. analysis using NRG-GY018 data estimated ICERs of approximately $41,000/QALY in dMMR disease and $90,000/QALY in pMMR disease, classifying pembrolizumab plus chemotherapy as cost-effective at $150,000/QALY (26). A later U.S.-China model with a five-year horizon reported a more conservative result: the regimen was economically favorable for dMMR patients in the United States but not for pMMR disease, and was not cost-effective in China at the assumed thresholds and prices (27). The contrast illustrates the sensitivity of results to time horizon, survival extrapolation, acquisition cost, and country-specific thresholds.

The pattern is clearer for dostarlimab. One analysis estimated an ICER near $60,000/QALY in dMMR disease but approximately $176,000/QALY in pMMR disease; a modest price discount was required for the pMMR result to reach the $150,000/QALY threshold (28). A separate evaluation found that atezolizumab plus chemotherapy was not cost-effective in either subgroup at current assumptions, but a two-year treatment cap reduced the dMMR ICER to an economically favorable range (29). This result is important because it identifies treatment duration as a value lever that can be altered without changing the companion diagnostic.

Durvalumab-based strategies have generally produced less favorable estimates. A DUO-E-based model reported ICERs above common U.S. thresholds for durvalumab with or without olaparib across dMMR and pMMR groups (30). A separate dMMR-focused analysis reached the same conclusion and identified durvalumab price as the principal driver (31). These findings do not negate clinical efficacy; they show that a highly responsive biomarker subgroup does not guarantee cost-effectiveness when maintenance therapy is prolonged or combined with another costly agent.

In previously treated disease, pembrolizumab plus lenvatinib improved survival compared with chemotherapy (32), but economic findings vary sharply by setting. A U.S.-based analysis estimated ICERs exceeding $378,000/QALY overall and $413,000/QALY in pMMR disease (33), whereas a Swedish analysis concluded that the regimen was cost-effective under local inputs and thresholds (34). Cross-country differences in negotiated prices, utility values, subsequent-treatment costs, and willingness-to-pay thresholds prevent transfer of a single ICER between health systems.

## 5 Patient selection beyond binary MMR status

MMR is currently the strongest practical predictor, but it is not a perfect binary gate. In the first-line setting, pMMR patients can derive benefit from anti-PD-1 combinations, although average gains are smaller and economic uncertainty is greater (12, 13, 18). Conversely, some dMMR tumors do not respond. Biomarker analyses suggest that TMB, PD-L1 expression, and the etiology of MMR deficiency may further refine response probabilities (20, 21). More broadly, emerging cellular immunotherapy strategies, including CAR-NK approaches, reinforce the importance of biomarker-informed patient selection (35, 36), but no secondary marker is yet sufficiently validated to replace MMR in routine selection.

pMMR disease is particularly heterogeneous. POLE mutations, TP53 abnormalities, homologous-recombination alterations, immune-cell density, and histologic subtype may create clinically distinct subgroups. Exploratory analyses have shown extensive overlap of PD-L1, TP53, BRCA1/2, and homologous-recombination biomarkers within pMMR tumors (17). From an economic perspective, this is a value-of-information problem: additional testing is worthwhile only when it changes treatment choice enough to offset its cost and the consequences of misclassification. A broad panel that produces information without altering management adds cost but not value.

Accessible tissue biomarkers can also support pragmatic refinement. CD8-positive tumor-infiltrating lymphocytes correlate with MMR and p53 patterns and may provide prognostic information (37). NGS-derived MSI and TMB can complement IHC in selected cases and may better capture an immunogenic phenotype, although tumor-specific calibration remains necessary (38). The near-term goal should be a hierarchical model: MMR as the primary companion diagnostic, followed by targeted secondary testing in pMMR tumors or in clinically discordant dMMR cases.

## 6 Value-based implementation

A value-based MMR pathway begins at diagnosis. The pathology report should identify the lost protein pattern, internal controls, heterogeneous or subclonal staining, and whether reflex methylation or molecular confirmation is required. Turnaround time should be treated as an outcome because delayed results can postpone high-value therapy. Laboratories and oncology services should monitor test failure, repeat testing, discordance resolution, and the proportion of advanced patients with actionable results available before treatment.

Economic evaluation should model the test and therapy jointly. Relevant outcomes include total testing cost, time to result, proportion correctly classified, drug acquisition and administration, immune-related toxicity, subsequent treatment, QALYs, budget impact, and distributional effects. Published immunotherapy analyses often reach different conclusions because they use different time horizons, extrapolation functions, utility values, and prices (39). Transparent reporting according to CHEERS 2022 is therefore essential (40).

Several policy levers can improve value without restricting clinically appropriate care. These include negotiated prices, fixed treatment-duration caps, stopping rules for progression or toxicity, outcomes-based agreements, and reassessment when mature overall-survival data become available. Because MMR IHC is comparatively inexpensive and widely deployable, universal access may reduce inequity; however, reflex NGS and specialist pathology review may remain concentrated in high-resource centers. Regional networks and standardized referral pathways are needed so that economic efficiency does not come at the cost of unequal access.

## 7 Conclusions

Our view is that MMR-guided immunotherapy should be implemented as a linked diagnostic-therapeutic pathway rather than as a drug-only decision. Universal four-protein IHC with reflex MLH1 promoter methylation and selective orthogonal molecular testing offers a practical way to identify dMMR/MSI-H patients, in whom the combination of large clinical benefit and relatively low testing cost produces the strongest value proposition, while avoiding an uncritical assumption that every pMMR regimen is economically attractive. A strength of this Mini Review is its integration of molecular testing, pivotal biomarker-stratified efficacy data, and jurisdiction-specific economic evaluations, which clarifies how assay performance, price, treatment duration, survival extrapolation, and willingness-to-pay thresholds jointly determine value. Its limitations are the narrative rather than systematic design, the small and heterogeneous economic evidence base, dependence on modeled rather than trial-collected resource use and utilities, limited mature overall-survival data, and imperfect transferability of ICERs across health systems. Future research should prospectively collect resource use, quality-of-life, and equity outcomes within biomarker-stratified trials; validate secondary predictors for pMMR tumors; quantify the clinical and economic consequences of subclonal or discordant MMR results; and use value-of-information analyses to prioritize evidence generation. Reimbursement policies should be revisited as mature survival data, negotiated prices, and duration-limited treatment strategies emerge. In conclusion, MMR status is both a predictive biomarker and an allocation tool, but sustainable value will depend on diagnostic quality assurance, selective treatment, transparent economic modeling, and equitable access to confirmatory testing.
